# Supplementary material for: Patterns of Intron Gain and Loss in Fungi
Source: PLoS Biol. 2004 Nov 30;2(12):e422. doi: 10.1371/journal.pbio.0020422 (PMC532390; doi:10.1371/journal.pbio.0020422)
Supplement: Table S1 — Also available at http://genes.mit.edu/NielsenEtAl/. (4.3 MB ZIP). [file pbio.0020422.st001.zip › NielsenEtAl/html/628.html]

AN5950.1.NCU05232.1.MG04150.1.FG09735.1


```
 CLUSTAL W (1.82) Multiple Sequence Alignments - Introns Inserted


Sequence 1: MG04150.1	724 aa
Sequence 2: FG09735.1	713 aa
Sequence 3: NCU05232.1	726 aa
Sequence 4: AN5950.1	723 aa
Alignment Length: 758 aa
Number Identitical Residues: 444 aa
Alignment Score (without introns) 19203


MG04150.1 	-MSAPQGGDSK----LFAR0GKVAELRLELNSGGKKDKNFTAKKIALKKIVANMTMSNND
NCU05232.1	MSAAQGGGDSK----LFAR0GKVAELRLELNSGSKKDKNFTQKKIALKKIVANMTMSNND
FG09735.1 	MSSAAGGGDSK----LFAR0GKVAELRLELNSGGKKDKNYAGKKIALKKIVANMTMSNND
AN5950.1  	MFIAPYVQSSLPPWVRTSQ~GKVAELRQELNSGGKKDKNYSAKKIALKKIVANMTMSNND
          	   *    .* ..    :: ******* *****.*****:: ******************

MG04150.1 	MVALFPDVIGCMHIESLEIKKM2CFLFLVNYARMRPEIAIKAIPVLEY0DMRDSNPLVRA
NCU05232.1	MVGLFPDIVACMAIQSLEIKKM~CFLYLVNYARMKPEIAVKAIPVLEH0DMEDPNPLVRA
FG09735.1 	MVALFPDIIGCMGIQSLEIKKM2CFLFLVNYARMRPEIAVKAIPVLQH0DMEDHNPLVRA
AN5950.1  	MIALFPDVIDCMNLPSLEIKKM2CFLFLVNYSRMKPDIALKALPILIN0DMEATNPLVRA
          	*:.****:: ** : ******* ***:****:**:*:**:**:*:*   **.  ******

MG04150.1 	LALRTMSYIHVREFVEATVPIVKQMLKDSDPYVRKTAAFCVAKLYDHDKQMVEQSDLIDR
NCU05232.1	LALRTMSYIHVREFVEATVPLVKHLLRDIDPYVRKTAAFCVAKLYDHDRHMVEASDLIDR
FG09735.1 	LALRTMSYIHVREFVEATVPIVKHMLKDGDPYVRKTAAFCVAKLYDHDRNMVESSDLIDR
AN5950.1  	LALRTIAYIHVREYVEATVQPLKRLMGDMDPYVRKTAAFCVAKLYEHDRKMVEASDLIDR
          	*****::******:*****  :*::: * ****************:**::*** ******

MG04150.1 	LNGLLRDDNPTVVASALASLMDIWERS~DAIKLTIDYGNASKMVAILPDCSE2WGQTYIL
NCU05232.1	LNNLLRDDNPTVVASALASLMDIWERT1----------------------SR~WGQTYIL
FG09735.1 	LNSLLRDDNPTVVASALAGLMDIWERS~DAIKLTIDYSNASKMVAILPDCSE2WGQTYIL
AN5950.1  	LNAMLKDENPTVVSSVLASLVDIWGRS~ETISLTIDYASASKLVSILPDCSE2WGQSYIL
          	** :*:*:*****:*.**.*:*** *: .: . : . ..::.  :  ...*. ***:***

MG04150.1 	EALMSYLPVDSGEALLLAERIAPRLSHSNSAVVLTCIRVILYLMNYISDQKQISALCRKL
NCU05232.1	EALMSYIPQESGEALLLAERIAPRLSHSNSSVVLTCIRVILYLMNYISDQKQISALCRKL
FG09735.1 	EALMSYVPQESGEAVLLAERISPRLSHSNSSVVLTCIRVILYLMNYIADQKQISALCRKL
AN5950.1  	EALMSYVPQDSAEALLLAERIAPRLSHQNSAVVLTSIRVILYLMNYIAEERHLTSLSKKL
          	******:* :*.**:******:*****.**:****.***********::::::::*.:**

MG04150.1 	SPPLVTLLAKGPEVQYLALRNALLILQRRPEVLRNDIRVFFCKYNDPIYVKVTKLELIFM
NCU05232.1	SPPLVTLLAKGPEVQYLALRNALLILQRRPEVLRNDIRVFFCKYNDPIYVKVTKLELIFM
FG09735.1 	SPPLVTLLAKGPEVQYLALRNALLILQRRPEVLRNDIRVFFCKYNDPIYVKVTKLELIFM
AN5950.1  	SPPLVTLLSKPPEVQYLVLRNAILILQKRPEVLRNDIRVFFCNYNDPIYVKVTKLELIFM
          	********:* ******.****:****:**************:*****************

MG04150.1 	LANERNIDEVLTELRE~YATEIDVHFVRKAVRAIGKLAIKIEPAAPRCIDLLLELVATKV
NCU05232.1	LANEKNIDEVLTELRE~YATEIDVHFVRKAVRAIGKLAIKIEPAAKRCINLLLELVSTKV
FG09735.1 	LATEDNIDEVLTELRE~YATEIDVHFVRKAVRAIGKLAIKIEPAARRCINLLLELVATKI
AN5950.1  	LTTKENISVVLAELRE2YATEIDVHFVRKAVRAIGKLAIKIESAAKQCIDCLLDLVNAKI
          	*:.: **. **:**** *************************.** :**: **:** :*:

MG04150.1 	TYIVQEATVVIRNIFRKYPNQYESIIGTLCEHLDSLDEPEAKAAMVWVIGQYASRIENSD
NCU05232.1	TYIVQEATVVIRNIFRKYPNQYESIIGTLCEHLDSLDEPEAKAAMVWVIGQYASRIENSD
FG09735.1 	TYIVQEATVVIRNIFRKYPNQYESIISTLCEHLDSLDEPEAKAAMVWVIGQYADRIENSD
AN5950.1  	PYIVQEATVVIRNIFRKYPNQYESIIGQVIQNIDDLDEPEAKAAVIWIIGQYADRIENSA
          	.*************************. : :::*.*********::*:*****.***** 

MG04150.1 	ALLEDFLYSFADEPVEVQLALLTATVKLFIQRPTKGQELVPKVLKWATEETDNPDLRDRA
NCU05232.1	ALLEDFLYSFAEEPVEVQLALLTATVKLFIQRPTKGQDLVPRVLKWATEETDNPDLRDRA
FG09735.1 	ALLEDFLYSFAEEPVEVQLALLTATVKLFIQRPTKGQELVPKVLKWATEETDNPDLRDRA
AN5950.1  	DLLQDYLATFHDETIEVQLSLLTATVKLFIQRPTKAQQLVPQVLKWCTEETDDPDLRDRG
          	 **:*:* :* :*.:****:***************.*:***:****.*****:******.

MG04150.1 	YMYWRLLSTDIAMAKQIVMGEKPPITAESERLDPATLEEMCLNVGTLATVYLKPVQTVFR
NCU05232.1	YMYWRLLSTDMESAKRIVMGEKPAITAESERLDPVTLEEMCLNVGTLATVYLKPVQTVFR
FG09735.1 	YMYWRLLSTDMNAARQIVMGEKPAITAESERLDSATLEEMCLNVGTLATVYLKPVQTVFR
AN5950.1  	YMYWRLLSSDPTTARQVVMGQKPPISAESEKLDNRTLEELCLNVGTLATVYLKPVQQVFR
          	********:*   *:::***:**.*:****:**  ****:**************** ***

MG04150.1 	SARPRRLLDSPALQRQLLPPGSGPLAPPGPYENNKSLSMFGQGGRPANVDPRD-------
NCU05232.1	NARPRKLPDSPCLQRPDYLDYS----TAHQLPNPKSLSQFGQGHQPSDFDPRVPGSRPAP
FG09735.1 	SARPRKLQDSPALQKQNLLVAG---------DSQKSISMFGNAGAATDIDPR--------
AN5950.1  	SARTRRLQYSPALQKRVEEYGS------NSWQFPAPPLSLNPVTSPTAATAT--------
          	.**.*:*  **.**:      .             .   :.    .:   .         

MG04150.1 	-GSRGNALDSSGGNLAQAVSDADAYFAGVG-------------AQAMANMRVE-ERD-TF
NCU05232.1	PTSSSNTQSQQGGNLAQAVHDADAYFTQQANQQAAVNDADAYFAQQMRQMQIQ-QPGDSF
FG09735.1 	--SRNPMSGDGQGNIAQAVSDADAYFSGIG-------------TQQMTPMHGD-QGDDVF
AN5950.1  	--SANGSASTPTGNTSAAISAADNYFNSVAVG-----------TQQMAALDLGGREDGGF
          	  * .   .   ** : *:  ** **   .             :* *  :   .. .. *

MG04150.1 	GGTSGVDMGGGGYVVNQYTPQTVLQPGQ------DNRNGDLLML
NCU05232.1	GGDAGHYGYGQGYVVSQSSAYQPGMGGMNGMGGMNGAQGDLLL-
FG09735.1 	GGGNGHSTG---YVVSAHAPQAVLQPTQG-----AGSNGDLLML
AN5950.1  	GGGGAPQTQ---YVVNQ-SQQQAYQPHLAG----GAATGELLLL
          	**  .       ***.  :          .        *:**:
```
